# Engineering the Bacterial Microcompartment Domain for Molecular Scaffolding Applications

**DOI:** 10.3389/fmicb.2017.01441

**Published:** 2017-07-31

**Authors:** Eric J. Young, Rodney Burton, Jyoti P. Mahalik, Bobby G. Sumpter, Miguel Fuentes-Cabrera, Cheryl A. Kerfeld, Daniel C. Ducat

**Affiliations:** ^1^Biochemistry and Molecular Biology, Michigan State University, East Lansing MI, United States; ^2^MSU-DOE Plant Research Laboratory, East Lansing MI, United States; ^3^Computational Sciences and Engineering, Oak Ridge National Laboratory, Oak Ridge TN, United States; ^4^Center for Nanophase Materials Sciences, Oak Ridge National Laboratory, Oak Ridge TN, United States; ^5^Molecular Biophysics and Integrated Bioimaging Division, Berkeley National Laboratory, Berkeley CA, United States

**Keywords:** scaffold, synthetic biology, bacterial microcompartment, shell proteins, BMC, spatial organization, metabolic engineering, self-assembly

## Abstract

As synthetic biology advances the intricacy of engineered biological systems, the importance of spatial organization within the cellular environment must not be marginalized. Increasingly, biological engineers are investigating means to control spatial organization within the cell, mimicking strategies used by natural pathways to increase flux and reduce cross-talk. A modular platform for constructing a diverse set of defined, programmable architectures would greatly assist in improving yields from introduced metabolic pathways and increasing insulation of other heterologous systems. Here, we review recent research on the shell proteins of bacterial microcompartments and discuss their potential application as “building blocks” for a range of customized intracellular scaffolds. We summarize the state of knowledge on the self-assembly of BMC shell proteins and discuss future avenues of research that will be important to realize the potential of BMC shell proteins as predictively assembling and programmable biological materials for bioengineering.

## Introduction

**FIGURE 1:**
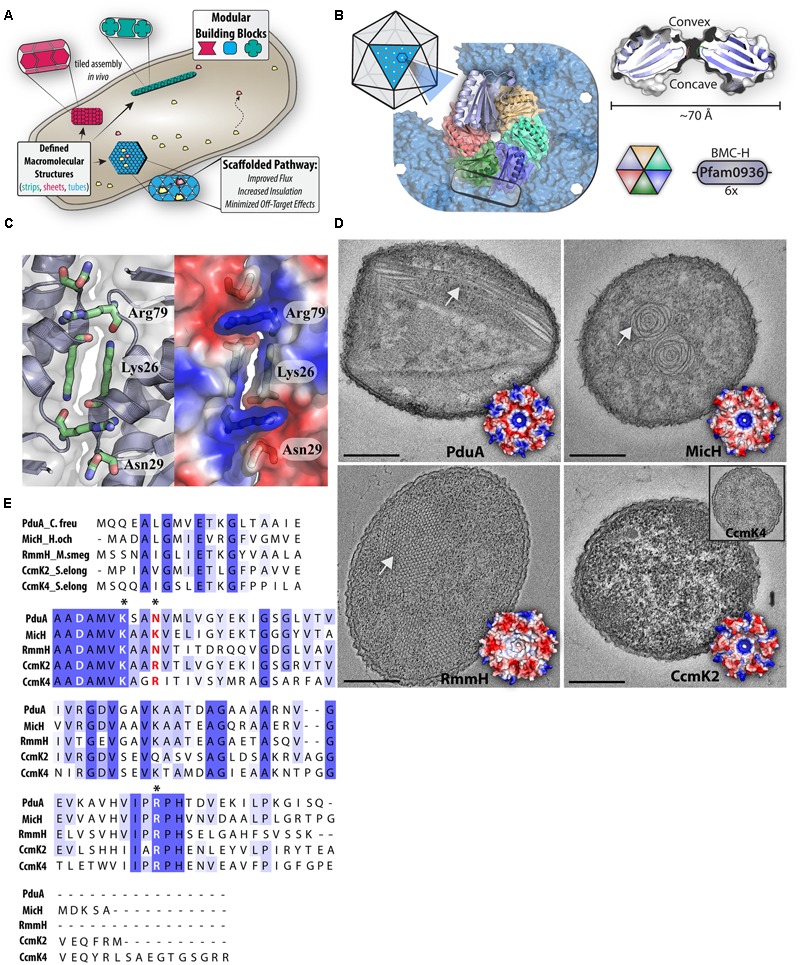
BMC-H attributes and potential as modular building blocks. **(A)** Cartoon schematic depicting distinct BMC hexamers (red, blue, and green) assembling into modular intracellular architectures that can recruit and concentrate cytosolic proteins (yellow and orange). **(B)** General features of BMC hexamers are highlighted through the example protein, PduA. A cross-section of a hexamer (right) illustrates the conserved shape and pore, while a hexamer is shown as part of a larger facet (left) that assembles through hexamer–hexamer contacts (box). **(C)** An expanded view of the interface between two BMC-H protein (PduA, PDB:3NGK), highlighting electrostatic interactions mediated by key residues (blue = positive). **(D)** Transmission electron microscopy of different assemblies of heterologously expressed BMC-H proteins in *E. coli* [PduA: Nanotubes (PDB:3NGK), MicH: Rosettes (PDB:5DJB), RmmH: Nanotubes (PDB:5L38), CcmK2: Lack of structure (PDB:4OX7)] Inlet: CcmK4. Scale bar 250 nm. **(E)** Multiple sequence alignment of representative BMC-H proteins. Asterisks indicate key residues positioned at the hexamer-hexamer interface.

With the advent of synthetic biology and recent advances in protein engineering, designing, constructing, and controlling biomolecule-based materials at the nanoscale is a rapidly developing field. Currently, there is a lack of modular building blocks for predictably fabricating custom sub-cellular architectures that can be subsequently programed with precise functions (**Figure 1A**). Because of their self-assembly properties, proteins containing the pfamdomain 00936 (pfam0936) are promising building blocks that can be repurposed to design novel protein scaffolds *in vivo*, distinct from their natural propensity to assemble bacterial microcompartments (BMCs). In this perspective, we discuss current research on pfam0936 proteins in the context of their potential as a biological material for the construction of custom nano-architectures and intracellular scaffolds.

Organizing interrelated cellular components in time and space is crucial to increase efficiency of diverse cellular processes, including processes in metabolism, signaling, and division (Pawson and Scott, 1997; Agapakis et al., 2012). Typically, cells colocalize components of a shared pathway, conferring a host of benefits that include increased enzymatic intermediate flux and limited pathway cross-talk (Good et al., 2011; Agapakis et al., 2012). Biological engineers have increasingly explored a variety of rational colocalization strategies to capitalize on such benefits. These engineered systems range in complexity from simple fusion proteins to dynamic artificial scaffolds (Conrado et al., 2008; Horn and Sticht, 2015; Myhrvold and Silver, 2015) or compartments (Giessen and Silver, 2016). As biologists move toward increasingly complex cellular engineering goals (Bashor et al., 2010), one challenge is designing sophisticated subcellular colocalization approaches that recapitulate the elegance of natural systems (Good et al., 2011). We focus here on molecular scaffold construction.

Polymerizing biomolecules represent ideal building blocks because they can self-assemble into higher-order arrangements *in vivo.* To date, DNA hybridization nanotechnology (e.g., DNA origami) is perhaps the best developed molecular building platform (Pinheiro et al., 2011). DNA architectures are especially flexible under non-physiological conditions where a nearly limitless array of architectures can be predictively constructed and controlled at scales approaching the sub-nanometer (Wilner et al., 2009; Fu et al., 2012; Funke and Dietz, 2016). Yet translating this technology to intracellular application has been partially constrained because the concentration of single-stranded nucleic acid building blocks and environmental properties important for nucleic acid folding (e.g., temperature, ions) are not easily manipulated *in vivo* (Pinheiro et al., 2011). While recent studies continue to advance the capability of nucleic acid assemblies achieved within the cell (Conrado et al., 2012; Myhrvold and Silver, 2015; Siu et al., 2015; Elbaz et al., 2016), proteins may offer another viable, naturally inspired solution. One early example of a synthetically designed scaffold was comprised of a string of protein–protein interaction domains that were used to recruit three cognate enzymes involved in the conversion of acetyl-CoA to mevalonate (Dueber et al., 2009). Co-recruitment of these enzymes substantially increased the mevalonate yield *in vivo*, yet only marginal improvements were reported when this approach was used for other metabolic pathways (Horn and Sticht, 2015). One proposed reason that this strategy is not widely successful is that this design lacks an inherent organized structure and may aggregate in unpredictable ways, hindering a rational design process (Lee et al., 2012). A genuinely modular protein-based scaffold would be composed of defined subunits which self-assemble into a concrete structure, which is dependent on the given application.

Toward this goal, engineering naturally found proteins which self-assemble into defined, nano to macromolecular architectures offers a powerful base to approach artificial scaffold construction (Howorka, 2011); the components of BMCs are particularly promising in this regard (Kerfeld and Erbilgin, 2015). In their native context, BMCs encapsulate related enzymes within a unique self-assembled protein shell (Kerfeld et al., 2010; Yeates et al., 2010; Axen et al., 2014; Kerfeld and Erbilgin, 2015). Many recent efforts have emphasized the engineering of BMCs to encapsulate new pathways for improved function of heterologous metabolic production (Bonacci et al., 2012; Choudhary et al., 2012; Lawrence et al., 2014; Cai et al., 2015; Gonzalez-Esquer et al., 2015; Lee et al., 2016; Quin et al., 2016; Baumgart et al., 2017; Liang et al., 2017; Slininger et al., 2017; Wagner et al., 2017; Yung et al., 2017). While BMCs hold much promise as defined, engineered compartments, their pfam0936 domain containing shell proteins are unique on their own and possess the capacity to self-assemble into a variety of higher-order structures when expressed in isolation (Havemann et al., 2002; Kerfeld et al., 2005; Parsons et al., 2008, 2010; Dryden et al., 2009; Pitts et al., 2012; Lassila et al., 2014; Pang et al., 2014; Noël et al., 2015; Sutter et al., 2015; Held et al., 2016). Loci encoding BMC-domain proteins are found in at least 23 bacterial phyla, while each instance having a minimal of three unique pfam0936 domain containing proteins (Axen et al., 2014). This diversity likely includes many new “building blocks” for constructing a multitude of novel, programmable architectures, but unlocking the true potential of the pfam0936 domain will require a deeper understanding of the fundamentals governing self-assembly. We propose that the establishment of design principles—rules which result in a defined, predictable assembly—for the pfam0936 domain will provide the foundation for creating an array of nano to macromolecular structures. These designer structures may then be functionalized to cater to their individual application (**Figure 1A**). We discuss the promise and potential limitations of this strategy below.

## Structural Characteristics and Self-Assembly of BMC-H Proteins

Numerous crystal structures of pfam0936-containing proteins have contributed to a detailed structural understanding of BMC shell proteins and models of how they “tile” into the facets of BMCs (**Figure 1B**) [summarized in Kerfeld and Erbilgin (2015)]. The signature domain of BMCs has little structural variation across the multitude of functionally distinct and distantly related BMCs, indicating a pivotal role in assembling the BMC shell (Crowley et al., 2010; Kinney et al., 2011). The main constituent of BMC shells are typically small (∼100 amino acids) proteins containing the BMC domain (BMC-H) which form a ∼70 Å hexagonal disk with distinct faces and a circular pore in the center (**Figure 1B**; Kerfeld et al., 2010; Yeates et al., 2010) other components of BMC shells are BMC-T (containing a tandem fused copy of pfam0936) and BMC-P (pfam03319) proteins (Kerfeld and Erbilgin, 2015), but are not a focus of this perspective. The concave side of BMC-H proteins features a surface depression that can harbor both flexible extensions of the N and C protein termini, whereas the convex side has varied electrostatic properties across homologs (**Figure 1B**; Kerfeld et al., 2010; Yeates et al., 2010).

In many crystal structures, a subset of residues found along the edge periphery mediate an inter-hexamer hydrogen bond network, permitting tiled assembly of conjoined arrays (**Figure 1C**; Kerfeld et al., 2005; Tanaka et al., 2008; Klein et al., 2009; Crowley et al., 2010; Takenoya et al., 2010; Kinney et al., 2011; Pitts et al., 2012; Cai et al., 2015). These edge residues (DxxK, RPH) are widely conserved throughout BMC-H proteins and thus, imply a crucial role in maintaining hexamer–hexamer interactions (Kerfeld and Erbilgin, 2015). For example, in the crystal structure of a PduA lattice—a canonical example—the antiparallel association of two adjacent lysine residues mediates the bulk of the inter-hexamer association (**Figure 1C**; Crowley et al., 2010; Pang et al., 2014; Sinha et al., 2014). Although, the buried interaction surface area at the hexamer-hexamer interface is typically less than other protein-protein interfaces, it is likely that the multiplicative nature of the interaction (1 hexamer surrounded by six others) provides sufficient cooperativity to permit higher-order arrays (Crowley et al., 2010). Since tiling behavior with consistent inter-hexamer distances has been observed in BMC-H sheets by high-resolution microscopy techniques (Dryden et al., 2009; Sutter et al., 2015), the interface observed in crystals is likely physiologically relevant.

The flexibility of high-order formation of BMC-H homologs begins to take shape outside the confining context of a crystalline array. Many distinct architectures can be formed by purified BMC-H proteins *in vitro*, including: 100 nm spheroids (Kerfeld et al., 2005; Keeling et al., 2014), extended nanotubes (Noël et al., 2015), and honeycombed tiles (Lassila et al., 2014). The macromolecular assembly behavior and the formation of such high-order structures are influenced by pH and ionic strength (Dryden et al., 2009; Noël et al., 2015; Jorda et al., 2016). Similarly, overexpression of BMC-H proteins *in vivo* leads to the self-assembly of a myriad of higher-order structures inside the cells, including: tubes (Parsons et al., 2010; Pang et al., 2014; Noël et al., 2015), filaments (Havemann et al., 2002; Parsons et al., 2008; Heldt et al., 2009; Pang et al., 2014), and other structures (Parsons et al., 2008, 2010; Pitts et al., 2012; Lin et al., 2014; Sutter et al., 2015; Held et al., 2016).

Because the methodology used to express BMC-H proteins varies among labs and studies (e.g., host, promoter strength, protein concentration, growth condition, sample preparation), it is not always clear if the distinct intracellular structures generated by BMC-H homologs in separate reports are due to intrinsic self-assembly properties, or the specific experimental conditions. Nonetheless, multiple lines of evidence suggest that properties of BMC-H proteins predispose them toward specific higher-order architectures (Pang et al., 2014; Sinha et al., 2014). To illustrate this point, we heterologously expressed a panel of BMC-H homologs from distinct BMCs under identical conditions in *E. coli* (Supplemental Material). We find that expression of BMC-H homologs PduA, MicH, RmmH, and CcmK2 form varied macromolecular assemblies *in vivo* (**Figure 1D**), generally in agreement with prior reports. PduA and RmmH form nanotube-like structures (Pang et al., 2014; Noël et al., 2015) and MicH (5815 BMC-H) forms “swiss roll” rosettes thought to be an extended sheet of rolled up protein (Sutter et al., 2015). Despite orderly tiling in CcmK2 and CcmK4 crystal structures (*Synechococcus elongatus* PCC 7942), over-expressing these homologs in *E. coli* does not lead to the formation of prominent macromolecular structures (**Figure 1D**). It is unclear if the absence of visible structures via transmission electron microscopy (TEM) thin section represents a lack of higher-order self-assembly, or if smaller assemblies are formed in the cytoplasm which are insufficiently discriminated from other cytoplasmic elements; as was previously proposed for other smaller BMC assemblies (Lassila et al., 2014). Collectively, it appears heterologously expressed BMC-H proteins form an assortment of *in vivo* assemblies, but exactly how the intrinsic features of each homolog (differences in primary structure) contribute to differences in self-assembly are currently unknown.

## Understanding the Determinants of Macromolecular Assembly of BMC-H Proteins

As evident by the diversity of structures formed by BMC-H proteins (**Figure 1D**), there must be subtle primary structure differences that dictate changes in higher-order assembly. One region anticipated to influence assembly dynamics surrounds residues at the inter-hexamer junction; although there is strict conservation of some sidechains at this interface, some positions exhibit variance across homologs (**Figure 1E**; Cai et al., 2015). For example, both CcmK2 and CcmK4 contain arginine in comparison to the asparagine residues of PduA, RmmH or lysine of MicH (**Figure 1E**, red text). Supporting this hypothesis, experimental evidence generated by targeted amino acid substitutions at interface residues of PduA indicate they influence macromolecular assembly *in vivo* (Pang et al., 2014). Other studies of BMC-H proteins with modified hexamer-hexamer interface residues show they alter the formation of isolated BMCs (Sinha et al., 2014), size of tiled arrays *in vitro* (Sutter et al., 2015), and disrupt crystal packing contacts and orientation (Pang et al., 2014; Sinha et al., 2014).

In addition to residues at the hexamer-hexamer interface, other hexamer features may dictate the self-assembly behavior of BMC-H homologs. It has been well documented that the overall electrostatic surface profiles varies significantly among homologs (**Figure 1D**; Kerfeld et al., 2010; Kinney et al., 2011). Electrostatic differences—known to effect the self-assembly of proteins (Keskin et al., 2008)—could influence the preferred interaction orientation between BMC-H proteins, predisposing them to a particular assembly architecture. Besides the overall electrostatic profile, other unique regions in primary structure of BMC-H homologs could manipulate self-assembly; one such region is the variable C-terminal region (**Figure 1E**, boxed). Longer C-terminal extensions (**Figure 1E**, CcmK4) originally were hypothesized to interfere with lateral molecular tiling through steric clash, a hypothesis partially supported by the observation of that truncation mutants form hexamers which pack more tightly in crystal lattices (Tanaka et al., 2009). Some crystal forms of CcmK2 orthologs appear to have hexamers which are stacked upon each other (dodecamer) interacting through the C-terminal extensions (Tanaka et al., 2009; Samborska and Kimber, 2012). Although the physiological significance of these crystal contacts is uncertain, *in vitro* analysis of the molecular weight (Tanaka et al., 2009) and FRET interaction (Samborska and Kimber, 2012) of CcmK2 with modified C-termini provide some supporting evidence of a functional role. However, it should be noted that C-terminal truncation does not disrupt the formation of heterologous BMC shells in the presence of other BMC shell components (Cai et al., 2016).

To establish detailed models for oligomeric BMC-H self-assembly, dynamic techniques that can interrogate the differences in the nucleation and expansion of shell protein arrays are required. High-speed atomic force microscopy (HS-AFM) is one emergent technique because of the high spatial and temporal resolution it affords. HS-AFM was used to capture the individual changes in BMC-H proteins association/dissociation rates into larger sheets (Sutter et al., 2015). Dynamic light scattering, and complimentary biophysical techniques can also quickly assess particle size (from single molecules to large assemblies) based on changes in the optical properties creating a more high-throughput pipeline to evaluate factors controlling assembly; as recently employed in a reengineered PduA variant (Jorda et al., 2016).

Computational frameworks and molecular dynamics offer another potentially powerful tool for predicting and understanding the behavior of BMC shell proteins. One recent study simulated the steps of BMC assembly by utilizing a computational model that varied the strength of hexamer-hexamer and hexamer-cargo affinity (Perlmutter et al., 2016). From this, two classes of BMC assembly emerged that proceeded through distinct hierarchies (Perlmutter et al., 2016). Another recent example used Monte Carlo simulations with a coarse-grained potential to study the 2D self-assembly of CcmK2 (*Synechocystis* sp PCC 6803) (Mahalik et al., 2016). In these simulations, 2D sheets were found to form rapidly after the association of an initial clustering of four hexamers, suggesting that self-assembly is rate-limited by a nucleation event (Mahalik et al., 2016). In turn, nucleation rates strongly depended on the concentration of hexamers and their relative 2D orientation upon collision (Mahalik et al., 2016).

**FIGURE 2:**
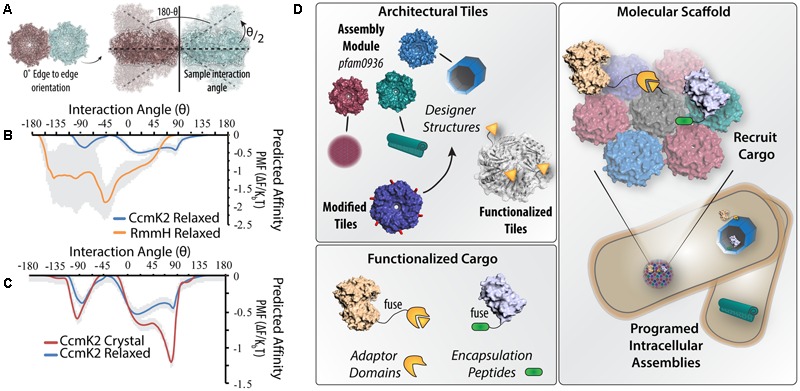
Could molecular-level simulations contribute toward predictive assembly of diverse BMC-H scaffolds? **(A)** Illustration of design of molecular dynamics simulations where the potential of mean force (PMF) is calculated from two adjacent hexamers. Keeping the relative orientation fixed, the hexamers are systematically rotated out of the plane by an angle 𝜃/2 and the change in PMF is recalculated. **(B)** Differences in the predicted PMF depending on the inter-hexamer angle are shown for solvated crystal structures of RmmH and CcmK2 (standard deviation between simulations is depicted in gray). **(C)** Differences in the angular PMF profile can solely arise by comparing the crystal structure versus solvated structure. **(D)** Illustration of pipeline for constructing BMC-H based programmable nanostructures. BMC-H proteins with different assembly characteristics can be selected from existing homologs (magenta, green, and blue) or created by modification of key residues (red) and modified to encode protein interaction domains (orange). Enzymes and other cargo can be directed to BMC-H assemblies by fusing corresponding ligand domains, or the use of native encapsulation peptides (green). In this manner, it is feasible to envision a diversity of subcellular protein architectures that can be functionalized to scaffold many distinct metabolic or signaling pathways.

To illustrate of how subtle differences in primary structure influences hexamers’ self-assembly, we performed preliminary simulations of the initial steps of the 3D self-assembly with two different BMC-H homologs (**Figures 2A–C**). Employing the Thomas-Dill (Thomas and Dill, 1996) coarse-grained potential—found to best approximate the fully atomic potential in Mahalik et al. (2016)—to compute the angular dependence in the potential of mean force (PMF) between two RmmH hexamers (*Mycobacterium smegmatis*) or two CcmK2 hexamers (*S. elongatus* 7942). The PMF was calculated as a function of the distance between the center of mass of each hexamer tile and the angle 𝜃 (defined in **Figure 2A**). CcmK2 and RmmH structures were obtained by relaxing the corresponding crystal structure in an aqueous environment (Supplemental Material). The angular dependence of PMF for CcmK2 and RmmH pairs is shown in **Figure 2B**; the PMF of RmmH has a clear minimum at 𝜃 ∼-45°, whereas CcmK2 is, by comparison, flatter. This minimum association angle of RmmH is consistent with the formation of regular repeating curved surface akin to the model created of *in vivo*/*in vitro* RmmH nanotubes (Noël et al., 2015). In contrast, *in vitro* macromolecular assemblies of CcmK2 orthologs (Keeling et al., 2014), depict flexibility in the overall macromolecular structure supporting a lack of defined interaction orientations. The differences in angular dependence of the PMF is further demonstrated if one uses different initial structures of the same BMC-H protein. This is illustrated in **Figure 2C** for CcmK2. In this figure, the PMFs of CcmK2 pairs formed from either the crystal or the relaxed structure are shown. The PMF of the crystalline pair has a clear angular minimum (𝜃 ∼-80°), where the PMF of the relaxed pair lacks any clear minimum. This is notable because the backbone root mean-square deviation between the two structures only differs by 0.67 Å. The distinct PMFs are caused by differences in side-chain rotamers altered in the relaxation procedure. These small structural changes are enough to cause a difference of almost 1 K_B_T, indicating that subtle structural changes which may occur between crystallization and *in situ* conditions can have a profound impact on self-assembly; also the overall lower PMF values for CcmK2 suggests a net weaker hexamer–hexamer interaction, consistent with other predictions (Perlmutter et al., 2016).

Although these observations of hexamer behavior in computational frameworks are preliminary, they are illustrative of the potential for *in silico* techniques to aid experimental methods in understanding and predicting behavior across several scales (from single molecules to large arrays). It is also noteworthy to mention the synergy between crystallography and electron microscopy for solving the macromolecular interactions in whole BMC assemblies (Sutter et al., 2017).

## Functionalization of Existing Macromolecular Structures

The engineering of scaffolding platforms based on self-assembling protein modules can be considered a two-faced challenge: one side is predictably making a discrete structure, and the other is functionalizing the structure for a specific purpose. Ideally, functionalizing the surface of BMC-H protein architectures should be modular in itself, so that scaffold structures could be “repurposed” for new enzymes and pathways with minimal redesign. There are two ways in which functional proteins could be organized to BMC-H assemblies: through natural or synthetic motifs.

One approach to functionalizing heterologous assemblies is to use binding motifs BMCs natively employed to recruit cargo (Fan et al., 2012; Aussignargues et al., 2015). Frequently, BMC core proteins contain small peptides (∼20 amino acids) as extensions of the N- or C- termini that are necessary for encapsulation (Choudhary et al., 2012; Kinney et al., 2012; Kim and Tullman-Ercek, 2014; Lawrence et al., 2014; Lin et al., 2014; Gonzalez-Esquer et al., 2015; Jakobson et al., 2015; Cai et al., 2016; Held et al., 2016; Quin et al., 2016; Wagner et al., 2017). Collectively known as “encapsulation peptides” (EPs), modeling (Fan et al., 2012; Kinney et al., 2012) and solution structures (Lawrence et al., 2014) adopt an alpha-helical conformation with an amphipathic charge distribution (Aussignargues et al., 2015). Although EPs vary widely in primary structure, it has been demonstrated that non-native EPs can interact with non-cognate BMCs (Jakobson et al., 2015) and amino acid substitutions can alter the affinity for BMC encapsulation (Kim and Tullman-Ercek, 2014), likely through conservation of specific amphipathic characteristics. While promising, general use of EP motifs for predictive recruitment is currently impaired by uncertainty in the interface location and affinity EP-BMC component binding (Fan et al., 2012; Lawrence et al., 2014; Aussignargues et al., 2015).

An alternative strategy is appending natural or synthetically derived protein–protein interaction domains—known as adaptor domains—to BMC-H proteins. In this way, virtually any protein encoded with the cognate adaptor domain could be post-translationally concentrated, conferring specified enzymatic functions to the designer architecture. While this approach is potentially powerful, it must be determined if fusion of adaptor domains to BMC-H proteins will alter higher-order assembly. In published work, fusions to BMC-H proteins have ranged from small affinity tags (Havemann et al., 2002; Kerfeld et al., 2005; Dryden et al., 2009; Samborska and Kimber, 2012) to fluorescent proteins (∼26 kDa: ∼2X the size of a single pfam0936 domain), and these modified BMC-H proteins still incorporate into BMCs (Parsons et al., 2008, 2010; Savage et al., 2010; Cai et al., 2013, 2015; Cameron et al., 2013; Sun et al., 2016). Yet, fusions of certain characteristics (e.g., size, charge) could disrupt association through steric clash or compromised electrostatics, thereby changing higher-order behavior. Indeed, although a fluorescent protein fusion to major shell protein CcmK2 incorporates into functional BMCs (Cameron et al., 2013), an unmodified copy of the protein must also be present, as the fusion is unable to solely complement a full *ΔCcmK2* background; this is supported also by fluorescent protein fusions to CcmK2 aggregates and does not interact with other shell protein components (Lin et al., 2014). In contrast, it is notable that some shell proteins can still assemble functional BMCs without a native copy present (Parsons et al., 2010; Sun et al., 2016), suggesting that some fusions can be tolerated or other shell protein components can relieve the compromised function of the fusion (Chowdhury et al., 2016). So, it remains to be fully elucidated how specific fusions and how certain properties (e.g., fusion orientation — N- vs. C-termini — fusion size/charge) will alter self-assembly behavior.

## Future Applications and Closing Remarks

The potential to form a spectrum of unique, functionalized architectures from a single, modulated building block can be anticipated to be a powerful tool for the future of synthetic biology and nanotechnology. While alternative approaches to build synthetic scaffolds have been proposed (see Introduction), often they do not possess defined geometry (Lee et al., 2012) or are not yet fully compatible for *in vivo* applications (Pinheiro et al., 2011). In a larger assembly, individual pfam0936 domains are structurally well-defined, which facilitates prediction of the relative position of the recruited proteins. This enables a design-test-build model of reiterative engineering, while also increasing the chance that successful designs from one pathway can be reimplemented for others.

Furthermore, the ability to construct different pfam0936 domain architectures raises the possibility that specific geometries can be chosen to tailor them for a given scaffolding application (**Figure 2D**). In the simplest case, scaffolding proteins to single hexamers or very small tiled arrays would be expected to concentrate up to 6 proteins per hexamer into cytosolic microdomains, akin to the earliest synthetic scaffold designs (Dueber et al., 2009). Stepping up in size, structures with 1D or 2D geometries—filaments or large planar sheets—could be more appropriate for the colocalization of signal-transduction or redox pathways; likened to a macromolecular switchboard (Good et al., 2011). More complex, sheltered architectures (e.g., nanotubes) would likely allow for increased efficiencies for metabolic pathways by enabling substrate channeling between a series of enzymes – an effective, but not yet fully understood, approach to increase pathway flux and insulation (Bauler et al., 2010; Idan and Hess, 2013; Castellana et al., 2014; Wheeldon et al., 2016). It is worthwhile to note that tubes, or other partially enclosed geometries, can realize roles akin to pathway encapsulation within a compartment, but would not require the engineering of small molecule-specific transporters or pores because of their access to the cytosol. As an added degree of utilization, it is feasible to imagine scaffolding orthogonal pathways on opposing faces of a given architecture (e.g., both on the outside and lumen of nanotubes). As the field determines the elements responsible for positioning BMCs inside cells (Savage et al., 2010), the ability to transpose these components to spatially localize entire pfam0936 domain assemblies arises, further increasing the engineering specificity for application.

Broadly speaking, we have outlined the promise and hurdles inherent to the use of pfam0936 domain containing proteins as building blocks for designer scaffold assemblies. One strength of using this domain to build ordered protein assemblies rests on the potential to construct a multitude of different intracellular assemblies from one common, protein domain. Even though the majority of published examples of higher-order pfam0936 assemblies are in prokaryotes, a recent report has documented engineering of several distinct higher-order structures in plant chloroplasts (Lin et al., 2014) indicating the potential for this approach across a wide diversity of organisms. Once design principles can be established, ways to further modify and cater the self-assembly process can be applied (Luo et al., 2016). Coupling this with more general knowledge on the principles of multiprotein complex formation (Ahnert et al., 2015; Murugan et al., 2015; Glover and Clark, 2016) the pfam0936 domain holds promise to aid in ushering in an era of true *in vivo* nanometer scale molecular engineering for the design of programmable architectures.

## Author Contributions

EY conceived the project ideas, conducted experiments and analyzed data, and wrote and edited the manuscript. RB, JM, BS, and MF-C contributed project ideas and experiments related to the computational work, and wrote and edited the manuscript. CK and DD conceived project ideas and wrote and edited the manuscript.

## Conflict of Interest Statement

The authors declare that the research was conducted in the absence of any commercial or financial relationships that could be construed as a potential conflict of interest.

## References

[B1] Agapakis C. M. Boyle P. M. Silver P. A. (2012). Natural strategies for the spatial optimization of metabolism in synthetic biology. *Nat. Chem. Biol.* 8 527–535. 10.1038/nchembio.975 22596204

[B2] Ahnert S. E. Marsh J. A. Hernández H. Robinson C. V. Teichmann S. A. (2015). Principles of assembly reveal a periodic table of protein complexes. *Science* 350:aaa2245 10.1126/science.aaa2245 26659058

[B3] Aussignargues C. Paasch B. C. Gonzalez-Esquer R. Erbilgin O. Kerfeld C. A. (2015). Bacterial microcompartment assembly: The key role of encapsulation peptides. *Commun. Integr. Biol.* 8:e1039755 10.1080/19420889.2015.1039755 PMC4594438 26478774

[B4] Axen S. D. Erbilgin O. Kerfeld C. A. (2014). A taxonomy of bacterial microcompartment loci constructed by a novel scoring method. *PLoS Comput. Biol.* 10:e1003898 10.1371/journal.pcbi.1003898 PMC4207490 25340524

[B5] Bashor C. J. Horwitz A. A. Peisajovich S. G. Lim W. A. (2010). Rewiring cells: synthetic biology as a tool to interrogate the organizational principles of living systems. *Annu. Rev. Biophys.* 39 515–537. 10.1146/annurev.biophys.050708.133652 20192780 PMC2965450

[B6] Bauler P. Huber G. Leyh T. McCammon J. A. (2010). Channeling by proximity: the catalytic advantages of active site colocalization using brownian dynamics. *J. Phys. Chem. Lett.* 1 1332–1335. 20454551 10.1021/jz1002007 PMC2865391

[B7] Baumgart M. Huber I. Abdollahzadeh I. Gensch T. Frunzke J. (2017). Heterologous expression of the *Halothiobacillus neapolitanus* carboxysomal gene cluster in *Corynebacterium glutamicum*. *J. Biotechnol.* 10.1016/j.jbiotec.2017.03.019 [Epub ahead of print]. 28359868

[B8] Bonacci W. Teng P. K. Afonso B. Niederholtmeyer H. Grob P. Silver P. A. (2012). Modularity of a carbon-fixing protein organelle. *Proc. Natl. Acad. Sci. U.S.A.* 109 478–483. 10.1073/pnas.1108557109 22184212 PMC3258634

[B9] Cai F. Bernstein S. L. Wilson S. C. Kerfeld C. A. (2016). Production and characterization of synthetic carboxysome shells with incorporated luminal proteins. *Plant Physiol.* 170 1868–1877. 10.1104/pp.15.01822 26792123 PMC4775138

[B10] Cai F. Sutter M. Bernstein S. L. Kinney J. N. Kerfeld C. A. (2015). Engineering bacterial microcompartment shells: chimeric shell proteins and chimeric carboxysome shells. *ACS Synth. Biol.* 4 444–453. 10.1021/sb500226j 25117559

[B11] Cai F. Sutter M. Cameron J. C. Stanley D. N. Kinney J. N. Kerfeld C. A. (2013). The structure of CcmP, a tandem bacterial microcompartment domain protein from the β-carboxysome, forms a subcompartment within a microcompartment. *J. Biol. Chem.* 288 16055–16063. 10.1074/jbc.M113.456897 23572529 PMC3668761

[B12] Cameron J. C. Wilson S. C. Bernstein S. L. Kerfeld C. A. (2013). Biogenesis of a bacterial organelle: the carboxysome assembly pathway. *Cell* 155 1131–1140. 10.1016/j.cell.2013.10.044 24267892

[B13] Castellana M. Wilson M. Z. Xu Y. Joshi P. Cristea I. M. Rabinowitz J. D. (2014). Enzyme clustering accelerates processing of intermediates through metabolic channeling. *Nat. Biotechnol.* 32 1011–1018. 10.1038/nbt.3018 25262299 PMC4666537

[B14] Choudhary S. Quin M. B. Sanders M. A. Johnson E. T. Schmidt-Dannert C. (2012). Engineered protein nano-compartments for targeted enzyme localization. *PLoS ONE* 7:e33342 10.1371/journal.pone.0033342 PMC3299773 22428024

[B15] Chowdhury C. Chun S. Sawaya M. R. (2016). The function of the PduJ microcompartment shell protein is determined by the genomic position of its encoding gene. *Mol. Microbiol.* 101 770–783. 10.1111/mmi.13423 27561553 PMC5003431

[B16] Conrado R. J. Varner J. D. DeLisa M. P. (2008). Engineering the spatial organization of metabolic enzymes: mimicking nature’s synergy. *Curr. Opin. Biotechnol.* 19 492–499. 10.1016/j.copbio.2008.07.006 18725290

[B17] Conrado R. J. Wu G. C. Boock J. T. Xu H. Chen S. Y. Lebar T. (2012). DNA-guided assembly of biosynthetic pathways promotes improved catalytic efficiency. *Nucleic Acids Res.* 40 1879–1889. 10.1093/nar/gkr888 22021385 PMC3287197

[B18] Crowley C. S. Cascio D. Sawaya M. R. Kopstein J. S. Bobik T. A. Yeates T. O. (2010). Structural insight into the mechanisms of transport across the *Salmonella enterica* Pdu microcompartment shell. *J. Biol. Chem.* 285 37838–37846. 10.1074/jbc.M110.160580 20870711 PMC2988387

[B19] Dryden K. A. Crowley C. S. Tanaka S. Yeates T. O. Yeager M. (2009). Two-dimensional crystals of carboxysome shell proteins recapitulate the hexagonal packing of three-dimensional crystals. *Protein Sci.* 18 2629–2635. 10.1002/pro.272 19844993 PMC2821281

[B20] Dueber J. E. Wu G. C. Malmirchegini G. R. Moon T. S. Petzold C. J. Ullal A. V. (2009). Synthetic protein scaffolds provide modular control over metabolic flux. *Nat. Biotechnol.* 27 753–759. 10.1038/nbt.1557 19648908

[B21] Elbaz J. Yin P. Voigt C. A. (2016). Genetic encoding of DNA nanostructures and their self-assembly in living bacteria. *Nat. Commun.* 7:11179 10.1038/ncomms11179 PMC4838831 27091073

[B22] Fan C. Cheng S. Sinha S. (2012). Interactions between the termini of lumen enzymes and shell proteins mediate enzyme encapsulation into bacterial microcompartments. *Proc. Natl. Acad. Sci. U.S.A.* 109 14995–15000. 10.1073/pnas.1207516109 22927404 PMC3443165

[B23] Fu J. Liu M. Liu Y. Woodbury N. W. Yan H. (2012). Interenzyme substrate diffusion for an enzyme cascade organized on spatially addressable DNA nanostructures. *J. Am. Chem. Soc.* 134 5516–5519. 10.1021/ja300897h 22414276 PMC3319985

[B24] Funke J. J. Dietz H. (2016). Placing molecules with Bohr radius resolution using DNA origami. *Nat. Nanotechnol.* 11 47–52. 10.1038/nnano.2015.240 26479026

[B25] Giessen T. W. Silver P. A. (2016). Encapsulation as a strategy for the design of biological compartmentalization. *J. Mol. Biol.* 428 916–927. 10.1016/j.jmb.2015.09.009 26403362

[B26] Glover D. J. Clark D. S. (2016). Protein calligraphy: a new concept begins to take shape. *ACS Cent. Sci.* 2 438–444. 10.1021/acscentsci.6b00067 27504490 PMC4965849

[B27] Gonzalez-Esquer C. R. Shubitowski T. B. Kerfeld C. A. (2015). Streamlined construction of the cyanobacterial CO2-fixing organelle via protein domain fusions for use in plant synthetic biology. *Plant Cell* 27 2637–2644. 10.1105/tpc.15.00329 26320224 PMC4815102

[B28] Good M. C. Zalatan J. G. Lim W. A. (2011). Scaffold proteins: hubs for controlling the flow of cellular information. *Science* 332 680–686. 10.1126/science.1198701 21551057 PMC3117218

[B29] Havemann G. D. Sampson E. M. Bobik T. A. (2002). PduA is a shell protein of polyhedral organelles involved in coenzyme B12-dependent degradation of 1, 2-propanediol in *Salmonella enterica* serovar Typhimurium LT2. *J. Bacteriol.* 184 1253–1261. 11844753 10.1128/JB.184.5.1253-1261.2002 PMC134856

[B30] Held M. Kolb A. Perdue S. Hsu S. Y. Bloch S. E. Quin M. B. (2016). Engineering formation of multiple recombinant Eut protein nanocompartments in *E. coli*. *Sci. Rep.* 6:24359 10.1038/srep24359 PMC4827028 27063436

[B31] Heldt D. Frank S. Seyedarabi A. Ladikis D. Parsons J. B. Warren M. J. (2009). Structure of a trimeric bacterial microcompartment shell protein, EtuB, associated with ethanol utilization in *Clostridium kluyveri*. *Biochem. J.* 423 199–207. 10.1042/BJ20090780 19635047

[B32] Horn A. Sticht H. (2015). Synthetic protein scaffolds based on peptide motifs and cognate adaptor domains for improving metabolic productivity. *Front. Bioeng. Biotechnol.* 3:191 10.3389/fbioe.2015.00191 PMC4655305 26636078

[B33] Howorka S. (2011). Rationally engineering natural protein assemblies in nanobiotechnology. *Curr. Opin. Biotechnol.* 22 485–491. 10.1016/j.copbio.2011.05.003 21664809

[B34] Idan O. Hess H. (2013). Engineering enzymatic cascades on nanoscale scaffolds. *Curr. Opin. Biotechnol.* 24 606–611. 10.1016/j.copbio.2013.01.003 23357532

[B35] Jakobson C. M. Kim E. Y. Slininger M. F. Chien A. Tullman-Ercek D. (2015). Localization of proteins to the 1, 2-propanediol utilization microcompartment by non-native signal sequences is mediated by a common hydrophobic motif. *J. Biol.* 290 24519–24533. 10.1074/jbc.M115.651919 PMC4591832 26283792

[B36] Jorda J. Leibly D. J. Thompson M. C. Yeateset T. O. (2016). Structure of a novel 13 nm dodecahedral nanocage assembled from a redesigned bacterial microcompartment shell protein. *Chem. Commun.* 52 5041–5044. 10.1039/c6cc00851h PMC5081076 26988700

[B37] Keeling T. J. Samborska B. Demers R. W. Kimber M. S. (2014). Interactions and structural variability of β-carboxysomal shell protein CcmL. *Photosynth. Res.* 121 125–133. 24504539 10.1007/s11120-014-9973-z

[B38] Kerfeld C. A. Erbilgin O. (2015). Bacterial microcompartments and the modular construction of microbial metabolism. *Trends Microbiol.* 23 22–34. 10.1016/j.tim.2014.10.003 25455419

[B39] Kerfeld C. A. Heinhorst S. Cannon G. C. (2010). Bacterial microcompartments. *Annu. Rev. Microbiol.* 64 391–408. 10.1146/annurev.micro.112408.134211 20825353

[B40] Kerfeld C. A. Sawaya M. R. Tanaka S. Nguyen C. V. Phillips M. Beeby M. (2005). Protein structures forming the shell of primitive bacterial organelles. *Science* 309 936–938. 10.1126/science.1113397 16081736

[B41] Keskin O. Gursoy A. Ma B. Nussinov R. (2008). Principles of protein-protein interactions: what are the preferred ways for proteins to interact? *Chem. Rev.* 108 1225–1244. 10.1021/cr040409x 18355092

[B42] Kim E. Y. Tullman-Ercek D. (2014). A rapid flow cytometry assay for the relative quantification of protein encapsulation into bacterial microcompartments. *Biotechnol. J.* 9 348–354. 10.1002/biot.201300391 24323373

[B43] Kinney J. N. Axen S. D. Kerfeld C. A. (2011). Comparative analysis of carboxysome shell proteins. *Photosynth. Res.* 109 21–32. 10.1007/s11120-011-9624-6 21279737 PMC3173617

[B44] Kinney J. N. Salmeen A. Cai F. Kerfeld C. A. (2012). Elucidating essential role of conserved carboxysomal protein CcmN reveals common feature of bacterial microcompartment assembly. *J. Biol. Chem.* 287 17729–17736. 10.1074/jbc.M112.355305 22461622 PMC3366800

[B45] Klein M. G. Zwart P. Bagby S. C. Cai F. Chisholm S. W. Heinhorst S. (2009). Identification and structural analysis of a novel carboxysome shell protein with implications for metabolite transport. *J. Mol. Biol.* 392 319–333. 10.1016/j.jmb.2009.03.056 19328811

[B46] Lassila J. K. Bernstein S. L. Kinney J. N. Axen S. D. Kerfeld C. A. (2014). Assembly of robust bacterial microcompartment shells using building blocks from an organelle of unknown function. *J. Mol. Biol.* 426 2217–2228. 10.1016/j.jmb.2014.02.025 24631000

[B47] Lawrence A. D. Frank S. Newnham S. Lee M. J. Brown I. R. Xue W. F. (2014). Solution structure of a bacterial microcompartment targeting peptide and its application in the construction of an ethanol bioreactor. *ACS Synth. Biol.* 3 454–465. 10.1021/sb4001118 24933391 PMC4880047

[B48] Lee H. DeLoache W. C. Dueber J. E. (2012). Spatial organization of enzymes for metabolic engineering. *Metab. Eng.* 14 242–251. 10.1016/j.ymben.2011.09.003 21946160

[B49] Lee M. DeLoache W. C. Dueber J. E. (2016). Employing bacterial microcompartment technology to engineer a shell-free enzyme aggregate for enhance 1-2 propanediol production in *Escherichia coli*. *Metab. Eng.* 36 48–56. 10.1016/j.ymben.2016.02.007 26969252 PMC4909751

[B50] Liang M. Frank S. Lünsdorf H. Warren M. J. Prentice M. B. (2017). Bacterial microcompartment-directed polyphaste kinase promotes stable polyphosphate accumulation *E. coli*. *Biotechnol. J.* 12. 10.1002/biot.201600415 28105684

[B51] Lin M. T. Occhialini A. Andralojc P. J. Devonshire J. Hines K. M. Parry M. A. (2014). β-Carboxysomal proteins assemble into highly organized structures in Nicotianachloroplasts. *Plant J.* 79 1–12. 10.1111/tpj.12536 24810513 PMC4080790

[B52] Luo Q. Hou C. Bai Y. Wang R. Liu J. (2016). Protein assembly: versatile approaches to construct highly ordered nanostructures. *Chem. Rev.* 116 13571–13632. 10.1021/acs.chemrev.6b00228 27587089

[B53] Mahalik J. P. Brown K. A. Cheng X. Fuentes-Cabrera M. (2016). Theoretical study of the initial stages of self-assembly of a carboxysome’s facet. *ACS Nano* 10 5751–5758. 10.1021/acsnano.5b07805 26906087

[B54] Murugan A. Zou J. Brenner M. P. (2015). Undesired usage and the robust self-assembly of heterogeneous structures. *Nat. Commun.* 6:6203 10.1038/ncomms7203 25669898

[B55] Myhrvold C. Silver P. A. (2015). Using synthetic RNAs as scaffolds and regulators. *Nat. Publ. Group* 22 8–10. 10.1038/nsmb.2944 25565027

[B56] Noël C. R. Cai F. Kerfeld C. A. (2015). Purification and characterization of protein nanotubes assembled from a single bacterial microcompartment shell subunit. *Adv. Mater. Interfaces* 3:1500295.

[B57] Pang A. Frank S. Brown I. Warren M. J. Pickersgill R. W. (2014). Structural insights into higher order assembly and function of the bacterial microcompartment protein PduA. *J. Biol. Chem.* 289 22377–22384. 10.1074/jbc.M114.569285 24873823 PMC4139245

[B58] Parsons J. B. Dinesh S. D. Deery E. Leech H. K. Brindley A. A. Heldt D. (2008). Biochemical and structural insights into bacterial organelle form and biogenesis. *J. Biol. Chem.* 283 14366–14375. 10.1074/jbc.M709214200 18332146

[B59] Parsons J. B. Frank S. Bhella D. Liang M. Prentice M. B. Mulvihill D. P. (2010). Synthesis of empty bacterial microcompartments, directed organelle protein incorporation, and evidence of filament-associated organelle movement. *Mol. Cell.* 38 305–315. 10.1016/j.molcel.2010.04.008 20417607

[B60] Pawson T. Scott J. D. (1997). Signaling through scaffold, anchoring, and adaptor proteins. *Science* 278 2075–2080. 9405336 10.1126/science.278.5346.2075

[B61] Perlmutter J. D. Mohajerani F. Hagan M. F. (2016). Many-molecule encapsulation by an icosahedral shell. *eLife* 5:e14078 10.7554/eLife.14078 PMC4947392 27166515

[B62] Pinheiro A. V. Han D. Shih W. M. Yan H. (2011). Challenges and opportunities for structural DNA nanotechnology. *Nat. Nanotechnol.* 6 763–772. 10.1038/nnano.2011.187 22056726 PMC3334823

[B63] Pitts A. C. Tuck L. R. Faulds-Pain A. Lewis R. J. Marles-Wright J. (2012). Structural insight into the *Clostridium difficile* ethanolamine utilisation microcompartment. *PLoS ONE* 7:e48360 10.1371/journal.pone.0048360 PMC3483176 23144756

[B64] Quin M. B. Perdue S. A. Hsu S. Y. Schmidt-Dannert C. (2016). Encapsulation of multiple cargo proteins within recombinant Eut nanocompartments. *Appl. Microbiol. Biotechnol.* 100 9187–9200. 10.1007/s00253-016-7737-8 27450681

[B65] Samborska B. Kimber M. S. (2012). A dodecameric CcmK2 structure suggests β-carboxysomal shell facets have a double-layered organization. *Structure* 20 1353–1362. 10.1016/j.str.2012.05.013 22748766

[B66] Savage D. F. Afonso B. Chen A. H. Silver P. A. (2010). Spatially ordered dynamics of the bacterial carbon fixation machinery. *Science* 327 1258–1261. 10.1126/science.1186090 20203050

[B67] Sinha S. Cheng S. Sung Y. W. McNamara D. E. Sawaya M. R. Yeates T. O. (2014). Alanine scanning mutagenesis identifies an asparagine–arginine–lysine triad essential to assembly of the shell of the Pdu microcompartment. *J. Mol. Biol.* 426 2328–2345. 24747050 10.1016/j.jmb.2014.04.012 PMC4089897

[B68] Siu K.-H. Chen R. P. Sun Q. Chen L. Tsai S.-L. Chen W. (2015). Synthetic scaffolds for pathway enhancement. *Curr. Opin. Biotechnol.* 36 98–106. 10.1016/j.copbio.2015.08.009 26322735

[B69] Slininger L. Jakobson C. M. Tullman-Ercek D. (2017). Evidence for improved encapsulated pathway behavior in a bacterial microcompartment through shell protein engineering. *ACS Synth. Biol.* 10.1021/acssynbio.7b00042 [Epub ahead of print]. 28585808

[B70] Sun Y. Casella S. Fang Y. Huang F. Faulkner M. Barrett S. (2016). Light modulates the biosynthesis and organization of cyanobacterial carbon fixation machinery through photosynthetic electron flow. *Plant Physiol.* 171 530–541. 10.1104/pp.16.00107 26956667 PMC4854705

[B71] Sutter M. Faulkner M. Aussignargues C. Paasch B. C. Barrett S. Kerfeld C. A. (2015). Visualization of bacterial microcompartment facet assembly using high-speed atomic force microscopy. *Nano Lett.* 16 1590–1595. 10.1021/acs.nanolett.5b04259 26617073 PMC4789755

[B72] Sutter M. Greber B. Aussignargues C. Kerfeld C. A. (2017). Assembly principles and structure of a 6.5-MDa bacterial microcompartment shell. *Science* 356 1293–1297. 10.1126/science.aan3289 28642439 PMC5873307

[B73] Takenoya M. Nikolakakis K. Sagermann M. (2010). Crystallographic insights into the pore structures and mechanisms of the EutL and EutM shell proteins of the ethanolamine-utilizing microcompartment of *Escherichia coli*. *J. Bacteriol.* 192 6056–6063. 10.1128/JB.00652-10 20851901 PMC2976447

[B74] Tanaka S. Kerfeld C. A. Sawaya M. R. Cai F. Heinhorst S. Cannon G. C. (2008). Atomic-level models of the bacterial carboxysome shell. *Science* 319 1083–1086. 10.1126/science.1151458 18292340

[B75] Tanaka S. Sawaya M. R. Phillips M. Yeates T. O. (2009). Insights from multiple structures of the shell proteins from the beta-carboxysome. *Protein Sci.* 18 108–120. 10.1002/pro.14 19177356 PMC2708042

[B76] Thomas P. D. Dill K. A. (1996). An iterative method for extracting energy-like quantities from protein structures. *Proc. Natl. Acad. Sci. U.S.A* 93 11628–11633. 8876187 10.1073/pnas.93.21.11628 PMC38109

[B77] Wagner H. J. Capitain C. C. Richter K. Nessling M. Mampel J. (2017). Engineering bacterial microcompartments with heterologous enzyme cargos. *Eng. Life Sci.* 17 36–46. 10.1002/elsc.201600107 PMC6999380 32624727

[B78] Wheeldon I. Minteer S. D. Banta S. Barton S. C. Atanassov P. Sigman M. (2016). Substrate channelling as an approach to cascade reactions. *Nat. Chem.* 8 299–309. 10.1038/nchem.2459 27001725

[B79] Wilner O. I. Weizmann Y. Gill R. Lioubashevski O. Freeman R. Willner I. (2009). Enzyme cascades activated on topologically programmed DNA scaffolds. *Nat. Nanotechnol.* 4 249–254. 10.1038/nnano.2009.50 19350036

[B80] Yeates T. O. Crowley C. S. Tanaka S. (2010). Bacterial microcompartment organelles: protein shell structure and evolution. *Annu. Rev. Biophys.* 39 185–205. 10.1146/annurev.biophys.093008.131418 20192762 PMC3272493

[B81] Yung M. Bourguet F. A. Carpenter T. S. Coleman M. A. (2017). Re-directing bacterial microcompartment systems to enhance recombinant protein expression of lysis protein E from bacteriophage φX174. *Microb. Cell Fact.* 16:71 10.1186/s12934-017-0685-x PMC5405515 28446197

