# Supplementary material for: Engineering the Bacterial Microcompartment Domain for Molecular Scaffolding Applications
Source: Front Microbiol. 2017 Jul 31;8:1441. doi: 10.3389/fmicb.2017.01441 (PMC5534457; doi:10.3389/fmicb.2017.01441)
Supplement: Supplementary file 1 [file Data_Sheet_1.PDF]

Materials and Methods

Transmission Electron Microscopy

E. coli BL21 DE3 Arctic Express were transformed with pET11b harboring the BMC-H protein of interest (MicH, RmmH, PduA, CcmK2, CcmK4) and single colonies were picked from selective plates and grown overnight in liquid LB supplemented with antibiotic. Cells were then back diluted and grown to $OD_{600} = 0.8$ at 30˚C. Cells were induced with 100 µM IPTG, grown for 14 hours at 20˚C, and fixed in 2.5% paraformaldehyde/glutaraldehyde cacodylate buffer solution for 24 hours at 4˚C. Fixative was then washed away, the cells were stained with osmium tetraoxide, washed, and then stained with 2% uranyl acetate. Samples were dehydrated with a series of acetone steps, and then embedded in Spurr resin. Blocks were trimmed and ~40 nm sections were cut using an ultramicrotome. Sections were then post stained with 2% uranyl acetate and Reynolds lead citrate and imaged on a JEOL electron microscope.

Methodology for Computational Calculations

The crystal structure of the CcmK2 hexamer was obtained from the protein databank (PDBID:4OX7), while the RmmH crystal structure was determined through molecular replacement with *H. ochraceum* BMC-H structure 5DJB. For both hexamers delta-protonated histidine parameters were used; simulating buffer conditions of pH 8 with both proteins having no titratable residues at this pH value. Hydrogen atoms were added using the program visual molecular dynamics (VMD) plugin psfgen. Each hexamer was solvated in a 20 nm water cube with 100 mM NaCl, using TIP3P water molecules. All these MD simulations were performed with the NAMD program with the CHARMM22 force fields. The short-range cutoff distance for nonbonded interactions was set to 12 Å, while long-range electrostatic interactions were calculated through the particle mesh Ewald method using a grid spacing of 1.0 Å. Langevin dynamics and a Langevin piston algorithm were both used; keeping temperature at 310 K and pressure at 1 atm. Time steps of 2 fs were employed. This system was treated with two equilibration steps: 100 steps of minimization and 1 ns equilibration

A Coarse grain(CG) model for the hexameric subunit was created first, followed by orientation and distance dependent potential of mean force (PMF) calculation. Residue based CG modeling was performed on the available all atomistic protein subunit structure. In CG modeling, each residue is represented by a bead centered at its Cβ position (except for glycine, which is centered at the Cα position). Each subunit is taken to be a rigid body, where the constituent residues don't interact with themselves but with the residues of the neighboring subunits. The short-range pairwise interaction potential between the 20 different naturally occurring residues were taken from Table 1 of Ref [2]. The details of the form of interaction potential are described in Ref [1].

For PMF calculation, two variables were used, relative orientation (θ), and the distance between the center of mass (COM) of two subunits (d) of the same kind (Figure 1a). The alignment of the two subunits was kept parallel to each other ($\varphi_1 = \varphi_2 = 90^o$) (Figure 1b). The choice of keeping the subunits aligned parallel to each other is guided from the fact that in nature, these subunits are mostly found to be parallel aligned [Ref]. The choice of variable θ is guided by the fact that some of the BMC's form nanotubes while others form flat sheet like structures when isolated proteins are allowed to self-assemble in-vitro [Ref]. For a fixed θ, d is varied and the minimum of pairwise interaction potential between the protein units is recorded. The same process was repeated by varying θ and potential minimum (~ PMF minimum) versus θ are reported.

References

[1] Mahalik, J. P., Brown, K. A., Cheng, X. and Cabrera, M. F., *ACS Nano*, 2016, 10, 5751.
[2] Thomas, P. D and Dill, K. A, *Proc. Natl. Acad. Sci. U. S. A.*, 1996, 93, 11628.
[3] C. A. Kerfeld *et al.*, Protein structures forming the shell of primitive bacterial organelles. *Science*. **309**, 936–938 (2005).
